# Supplementary material for: Monocytic and granulocytic myeloid derived suppressor cells differentially regulate spatiotemporal tumour plasticity during metastatic cascade
Source: Nat Commun. 2017 Apr 6;8:14979. doi: 10.1038/ncomms14979 (PMC5384228; doi:10.1038/ncomms14979)
Supplement: Supplementary Information — Supplementary Figures [file ncomms14979-s1.pdf]

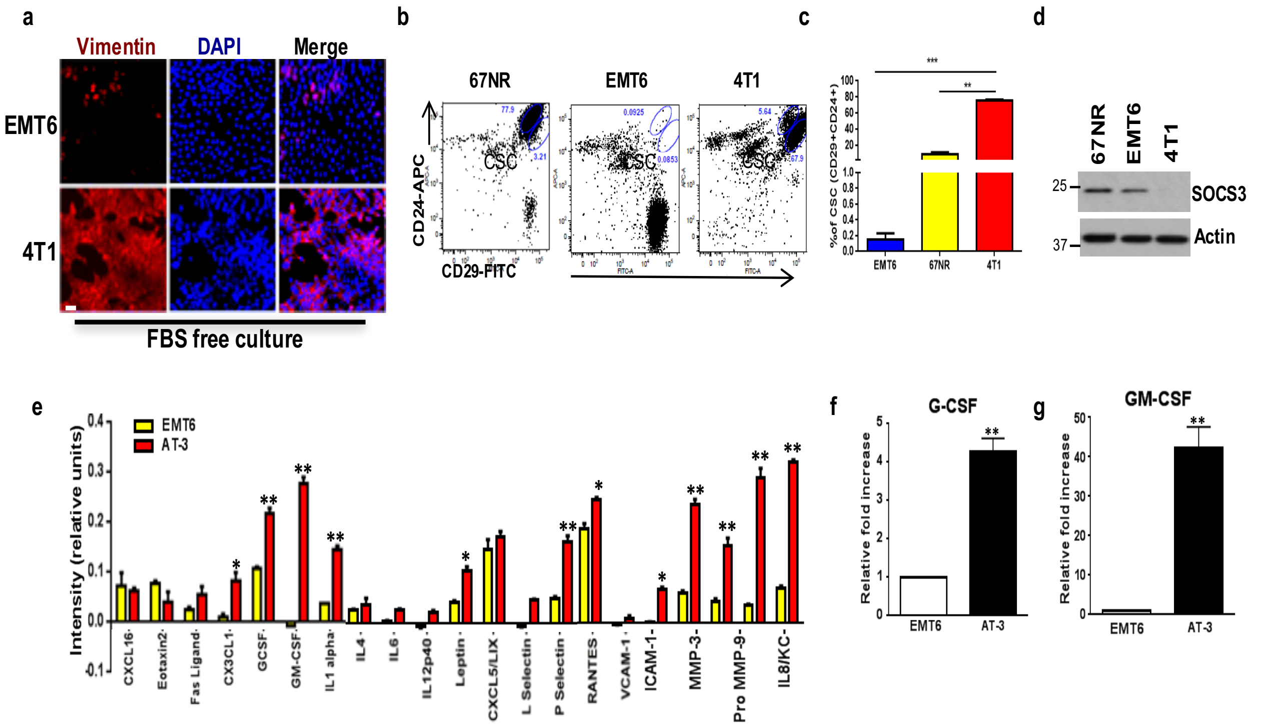

Supplementary Figure 1. 4T1 and AT-3 murine mammary tumor cells display tumorigenic and metastatic properties. (a) Metastatic 4T1 tumor cells show a higher Vimentin expression and **(b,c)** display higher cancer stem cell (CSC) phenotype as shown by CD24+CD29+ staining, compared to non-metastatic EMT6 or 67NR cells. **(d)** Western blot analysis shows the different expression of SOCS3 in non-metastatic 67NR, EMT6 and metastatic 4T1 cancer cells **(e)** AT-3 tumor cells secrete higher levels of inflammatory cytokines and growth factors as analyzed by antibody cytokine array. **(f, g)** Expression of G-CSF and GM-CSF genes in AT-3 cells are validated by qPCR. Scale bar 50µm *P<0.05, **P<0.005, ***P<0.0005, unpaired t-test.

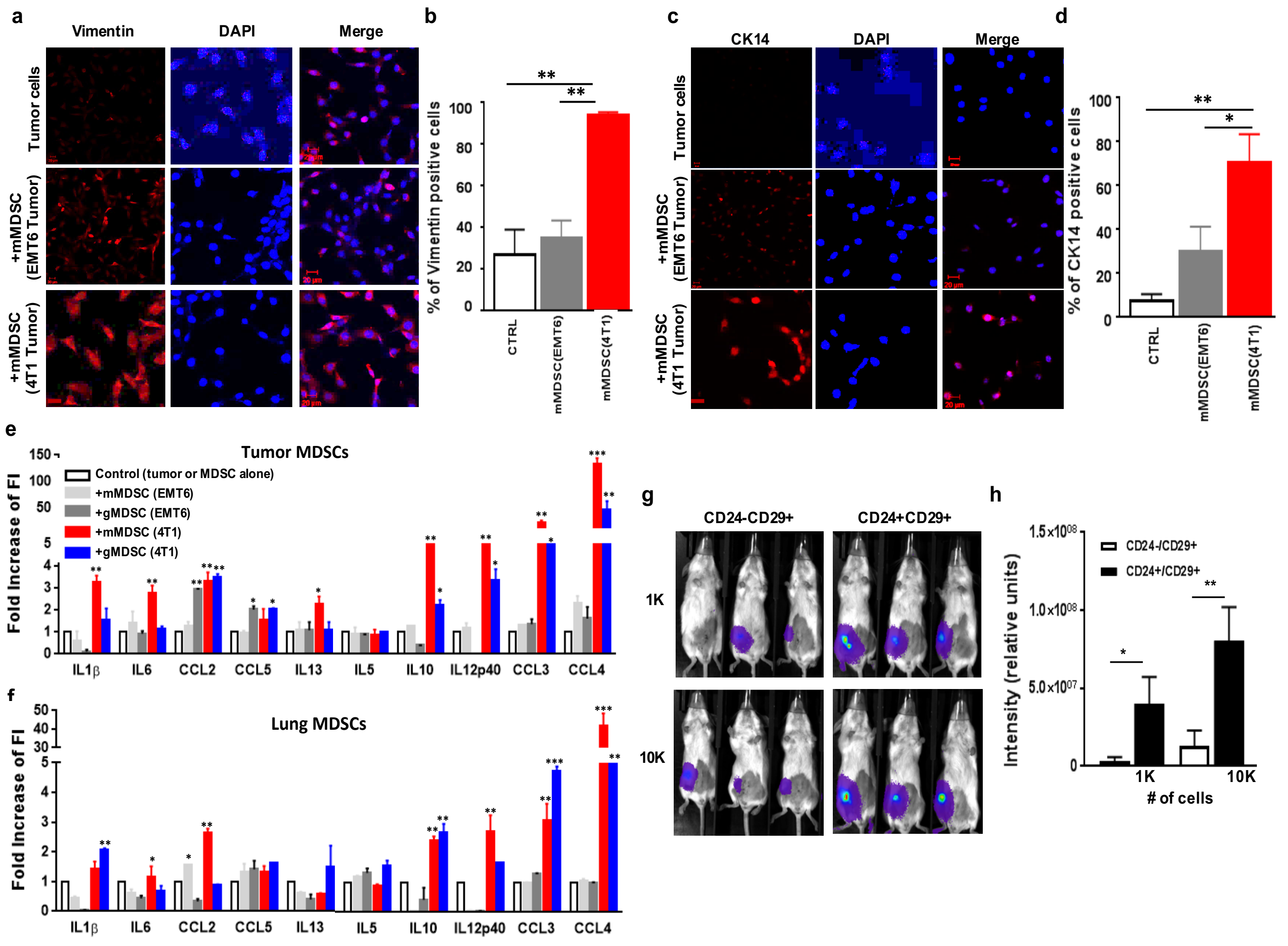

Supplementary Figure 2. MDSCs derived from 4T1 tumor bearing mice, compared to EMT6 tumor, are more potent in inducing aggressive phenotype in tumor cells. (a-d) mMDSCs from 4T1 tumor bearing mice, not from EMT6, enhance the expression of Vimentin and CK14 in tumor cells as assessed by immunofluorescence staining. **(e, f)** MDSC subsets from 4T1 tumor bearing animals induce wide range of cytokines in tumor cells as shown by multiplex assay. (g, h) mMDSC induced CSC (CD24+CD29+) population in EMT6 cells display a higher tumorigenic activity in mice compared to non-CSC (CD24-CD29+) population. Scale bar 50μm *P<0.05, **P<0.005, ***P<0.0005, unpaired t-test.

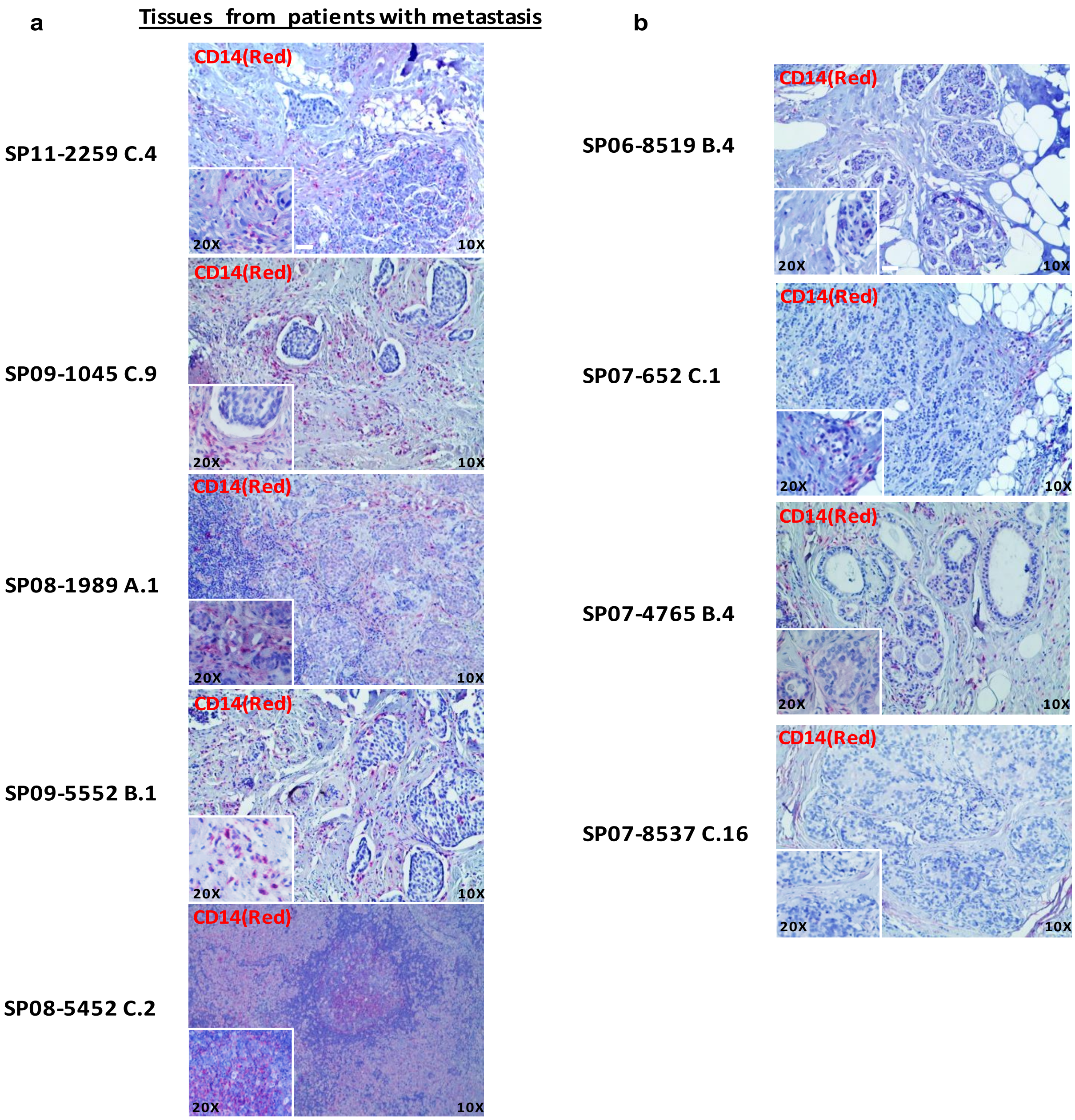

Supplementary Figure 3. High infiltration of CD14 positive cells is observed in metastatic human breast cancer tissues. Immunohistochemistry staining of 11 primary tumor samples using CD14 antibody shows that **(a)** higher number of CD14+cells are detected in metastatic tumors, **(b)** compared to the indolent tumors. Scale bar 50μm.

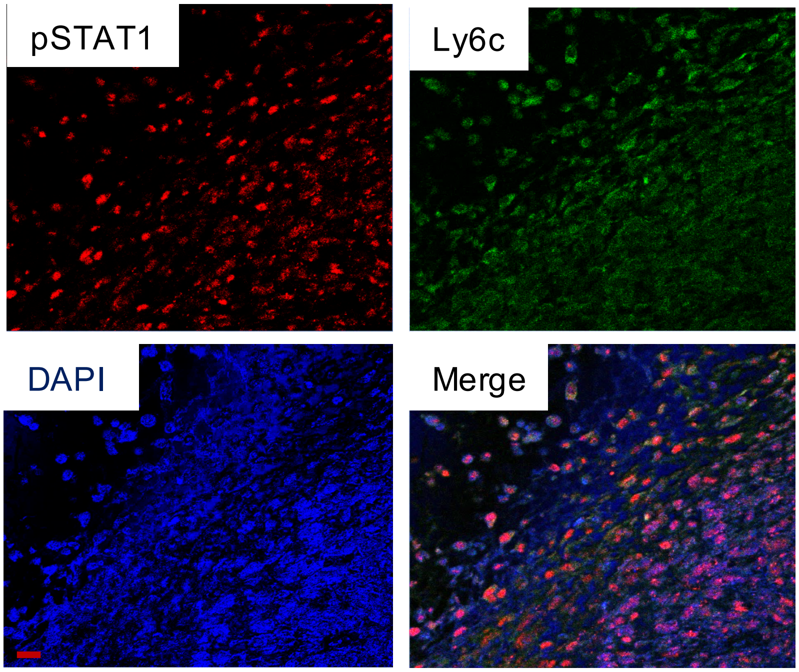

Supplementary Figure 4. Co-localization of pStat1 expressing tumor cells and Ly6C positive mMDSCs at tumor invasive front. 4T1 tumor xenografts at one-week post-implantation show elevated levels of pStat1-positive tumors co-localized with mMDSCs at the tumor invasive front. Scale bar 50µm.

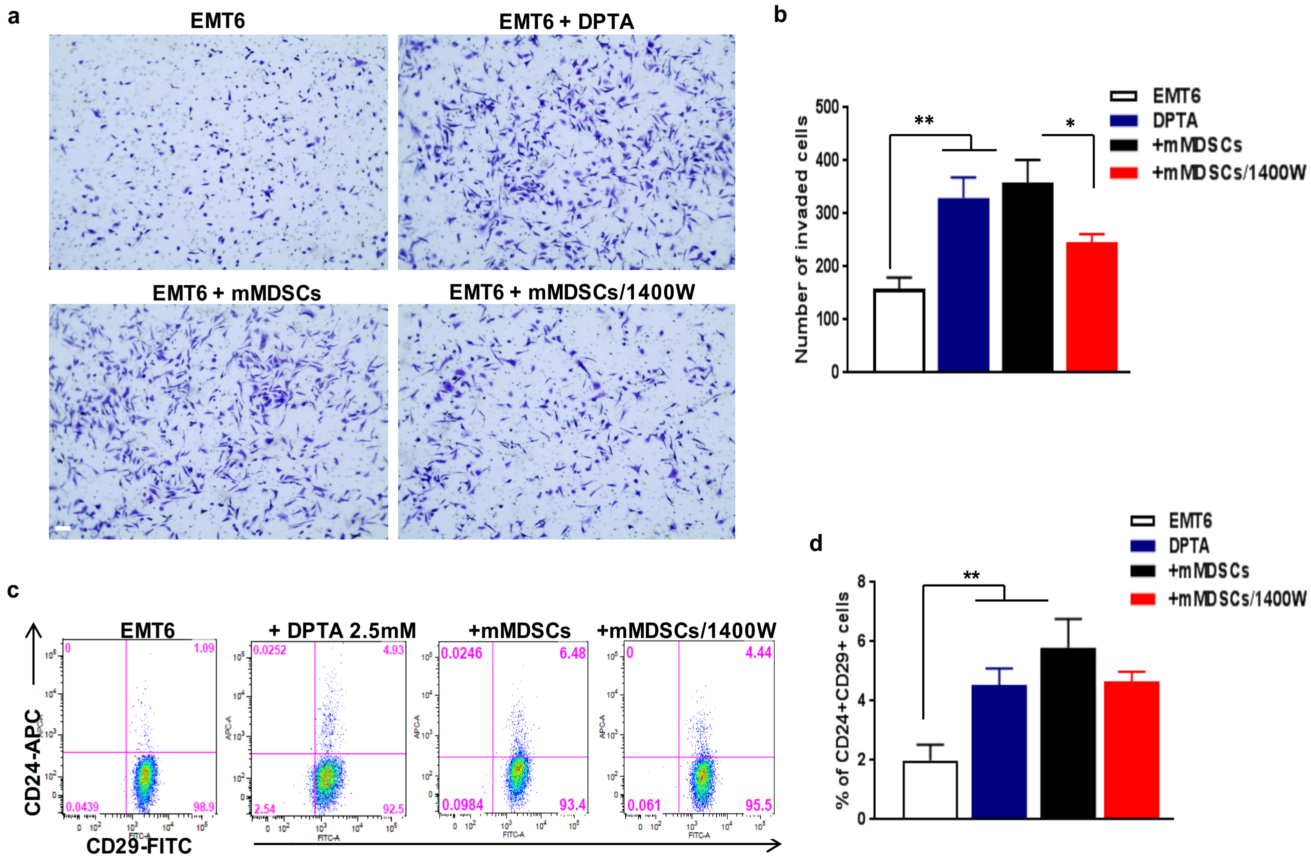

Supplementary Figure 5. mMDSC mediated NOS2 production regulate EMT/CSC phenotype and invasion in tumor cells. (a, b) Induction of NOS2 by DPTA donor promote tumor cell invasion (upper panels). Consistent with this, mMDSC induced tumor cell invasion was suppressed by NOS2 inhibitor, 1440W (lower panels). **(c, d)** NOS2 activation in tumor cells by DPTA increased the CSC population, while NOS2 inhibitor blocked the mMDCS induced CSC expansion. Tumor and lung derived gMDSCs from 4T1 tumor bearing mice highly enhance tumor cell proliferation compared to MDSCs isolated from EMT6 tumor bearing mice as shown by immunofluorescence staining of Ki67. Scale bar 50μm *P<0.05, **P<0.005, ***P<0.0005, unpaired t-test.

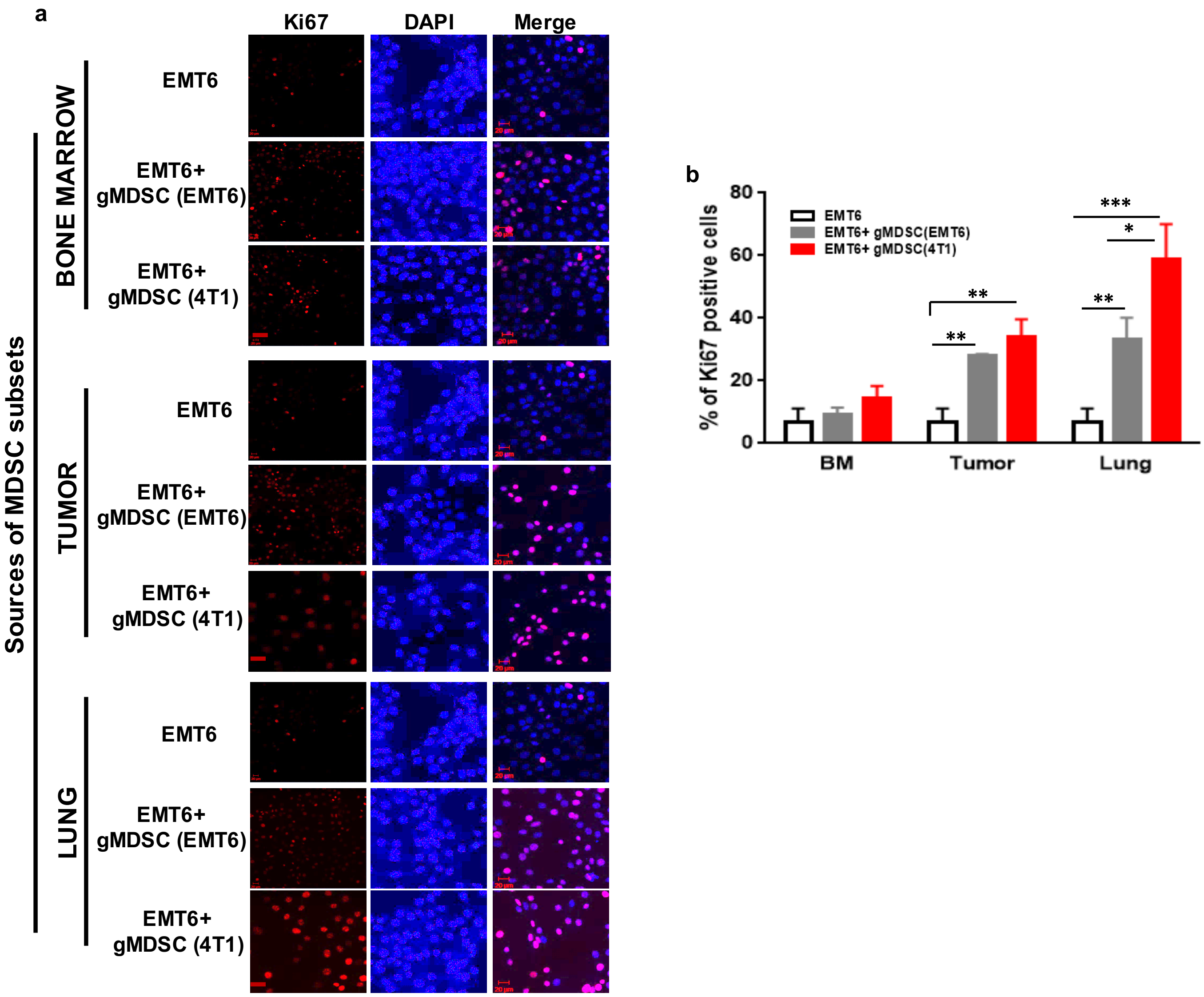

Supplementary Figure 6. gMDSCs derived from 4T1 tumor bearing mice, compared to EMT6 tumor, are more effective in promoting tumor cell proliferation. (a, b) Tumor and lung derived gMDSCs from 4T1 tumor bearing mice highly enhance tumor cell proliferation compared to MDSCs isolated from EMT6 tumor bearing mice as shown by immunofluorescence staining of Ki67. Scale bar 50μm *P<0.05, **P<0.005, ***P<0.0005, unpaired t-test.

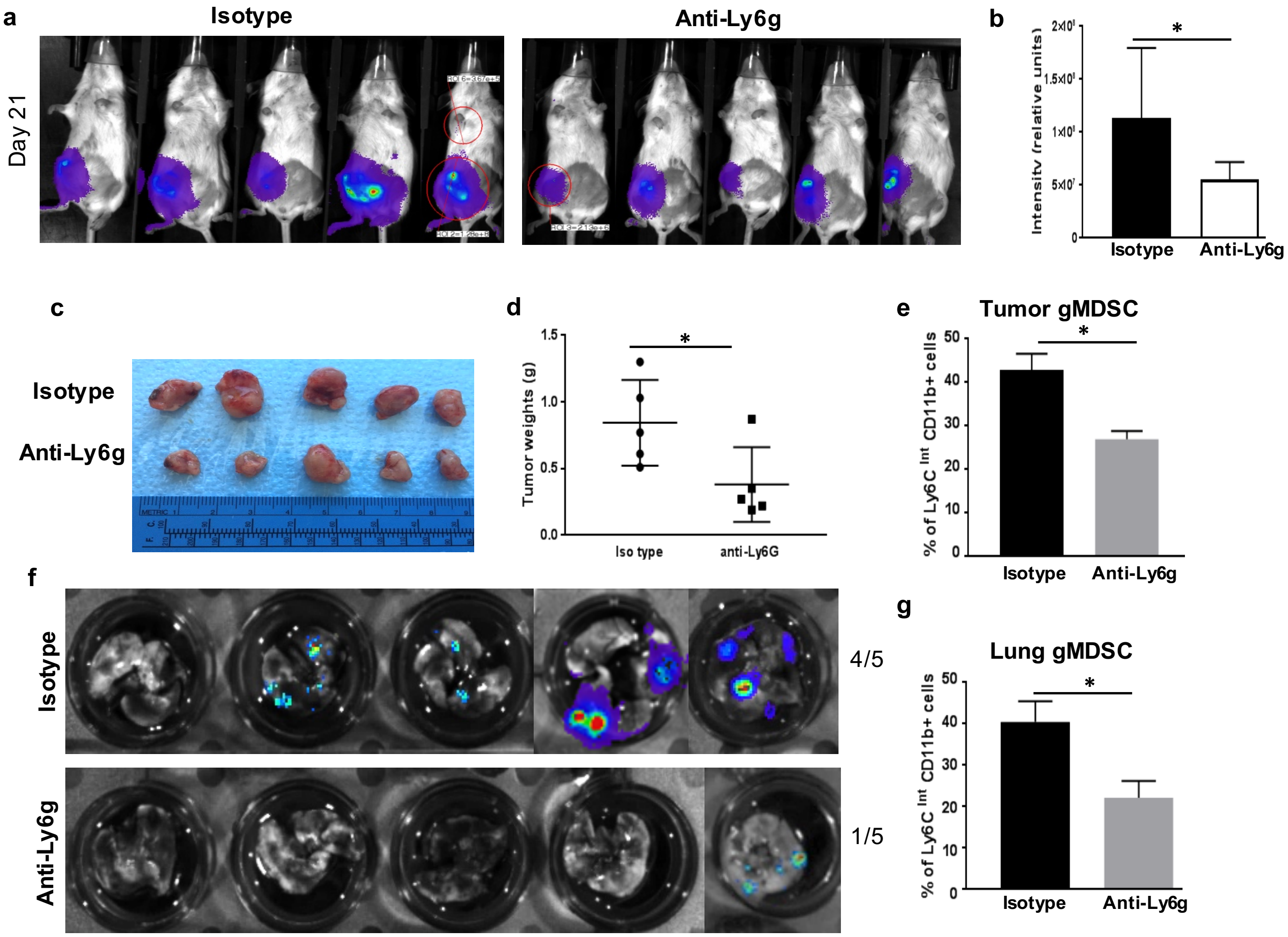

Supplementary Figure 7. Depletion of gMDSCs in 4T1 tumor-bearing mice suppress tumor growth and lung metastasis. **(a-d)** Treatment of 4T1 tumor-bearing mice with anti-Ly6g antibody (200μg three times a week for two weeks and thereafter 100μg) suppressed the tumor growth as well as **(f)** lung metastasis. **(e, g)** Suppression of tumor growth and lung metastasis may be due to reduced gMDSC infiltrates in primary tumor and lungs by Anti-Ly6g antibody. *P<0.05, unpaired t-test.

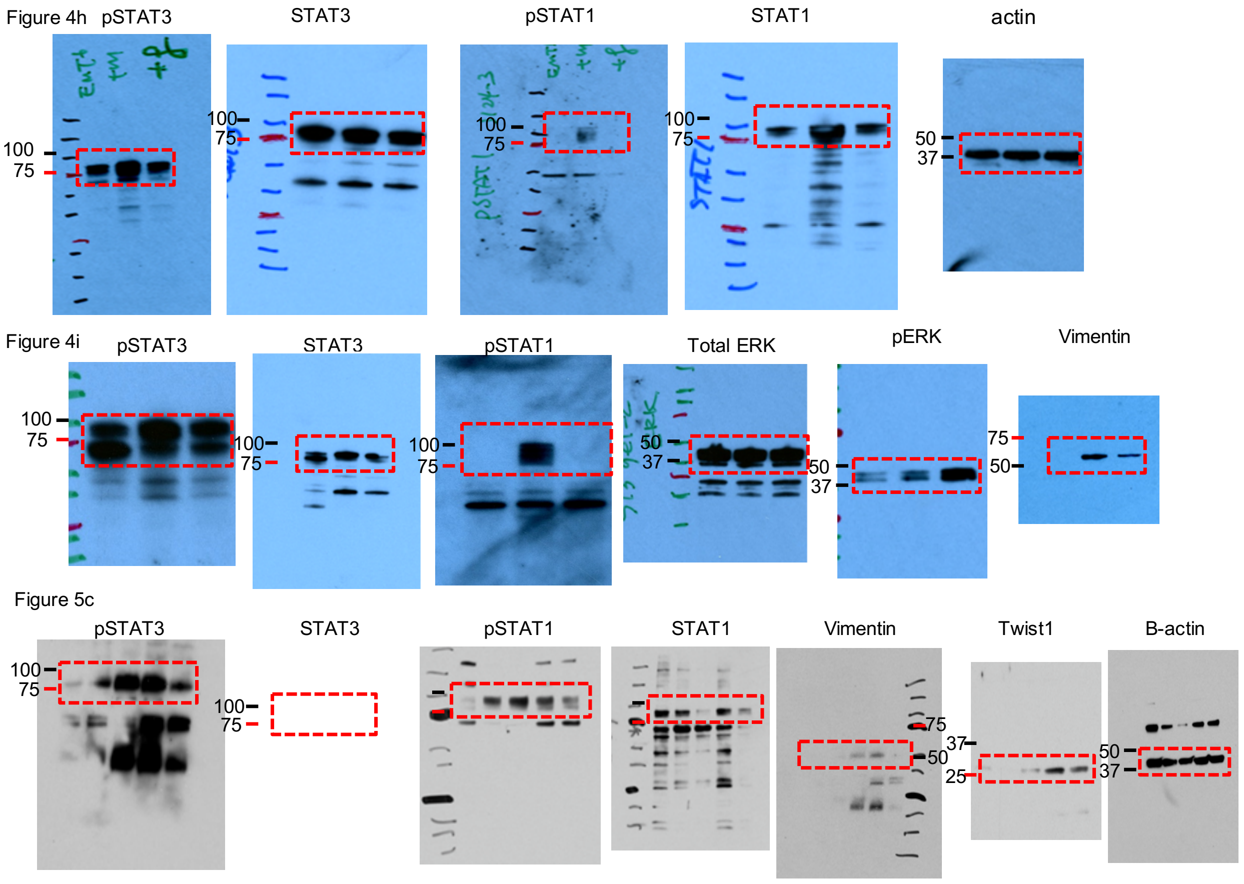

Supplementary Figure 8. Uncropped full western blot scans of important data in Figures 4 and 5.
